# Differential expression of plasma extracellular vesicles microRNAs and exploration of their association with bone metabolism in childhood trauma participants treated in a psychosomatic clinic

**DOI:** 10.3389/fendo.2025.1515910

**Published:** 2025-02-26

**Authors:** Yangyang He, Karin Wuertz-Kozak, Petra Cazzanelli, Sanne Houtenbos, Francisco Garcia-Carrizo, Tim J. Schulz, Pia-Maria Wippert

**Affiliations:** ^1^ Medical Sociology and Psychobiology, University of Potsdam, Potsdam, Germany; ^2^ Faculty of Health Sciences Brandenburg, Joint Faculty of the University of Potsdam, The Brandenburg, Medical School Theodor Fontane and The Brandenburg University of Technology Cottbus—Senftenberg, Potsdam, Germany; ^3^ Department of Biomedical Engineering, Rochester Institute of Technology, Rochester, NY, United States; ^4^ Schoen Clinic Munich Harlaching, Spine Center, Academic Teaching Hospital and Spine Research Institute of the Paracelsus Medical University Salzburg, Munich, Germany; ^5^ Department of Adipocyte Development and Nutrition, German Institute of Human Nutrition, Potsdam, Germany; ^6^ German Center for Diabetes Research (DZD), München, Germany; ^7^ Institute of Nutritional Science, University of Potsdam, Potsdam, Germany

**Keywords:** mental disorder, epigenetics, bone remodeling, osteoporosis, bone turnover markers

## Abstract

**Introduction:**

Early life stress (ELS) impacts neurotransmitters and cell communication, potentially disrupting neurological and physiological processes. Recently, ELS has been implicated in impaired bone metabolism, with extracellular vesicles (EVs) and their cargo, microRNAs (miRNAs), might affecting this process. This research aimed to elucidate the association between childhood trauma, a specific form of ELS, and bone metabolism through studying miRNA in EVs within three steps: firstly, examining alterations of EV miRNAs between ELS and controls, secondly analyzing associations between altered EV miRNAs and bone markers, and thirdly exploring the target gene prediction and enrichment pathways of altered EV miRNAs.

**Methods:**

This study included a subgroup of the DEPREHA project (total n=208) from a psychosomatic clinic. Firstly, real-time quantitative PCR was performed on plasma EVs isolated from childhood trauma participants with depression (n=6) and matched healthy controls (n=9) to detect the differentially expressed EV miRNAs. Secondly, general linear regression models were employed to investigate the associations between specific EV miRNAs and circulating bone turnover markers (procollagen type 1 amino-terminal propeptide (P1NP), osteocalcin, and β-CrossLaps (CTx)), adjusting for depression as a potential confounder. Thirdly, the miRNA target gene networks and enriched pathways were explored based on altered EV miRNAs.

**Results:**

These analyses could be conducted on n=19 participants from the entire group (11 [57.9%] female; median [IQR] age, 35.00 [26.00] years), but finally n=15 participants were included for analyses. 22 out 380 EV miRNAs were differentially expressed between childhood trauma participants (6 up-regulated and 16 down-regulated) and healthy controls. Among these, miR-25-3p, miR-26b-5p, miR-451a, and miR-421 were associated with P1NP (bone formation marker) and CTx (bone resorption marker). MiR-26b-5p, miR-330-3p, and miR-542-5p were associated with osteocalcin (bone turnover marker). MiRNA target gene network prediction revealed highly associated target genes of dysregulated miRNAs, such as Trinucleotide Repeat Containing Adaptor 6B (*TNRC6B*), and enrichment analysis highlighted pathways including the forkhead box protein O (FoxO) signaling pathway.

**Discussions:**

This study explored the potential associations between childhood trauma and bone metabolism, due to the sample size and experimental group limitations, these associations should be validated in future experiments with larger sample sizes and different control group settings.

## Introduction

Stress is a state in which the homeodynamic balance of an organism is threatened (1), and chronic or excessive stress reactions may cause a series of pathological reactions that affect the organism’s health. The term early life stress (ELS) refers to a single or a series of adverse events and stressful experiences during childhood, including childhood abuse, neglect, parental illness, separation, and poverty (2). As brain development largely occurs during this early stage of life, stressor exposure can have enduring effects, leading to the development of mental illness later in life (3, 4). Among adult patients suffering from mental illnesses, 53% report having experienced at least one childhood adversity (5), highlighting the high impact of ELS on public health.

ELS involves different moderating factors, including neuroendocrine stress responsiveness, the immune system, epigenetic programming, metabolism, and the transcriptome (6), which can induce biopsychological effects in later life. In this article, childhood trauma will be addressed, which represents a specific form of ELS and is defined as “a traumatic event is one that threatens injury, death, or the physical integrity of self or others and also causes horror, terror, or helplessness at the time it occurs.” (7). Accumulating evidence showed that childhood trauma is associated with various mental disorders in a lifetime, for example, posttraumatic stress disorder (8), anxiety (9), and depression (10). It has also been demonstrated that individuals with childhood trauma and during a depressive episode showed deteriorating effects on bone health, such as reduced bone mineral density, regardless of age (11). Previous studies have shown that depression is associated with increased bone loss (12) and fracture risk (13), and is considered a risk factor for osteoporosis (14–16). Bone metabolism consists of a balance between bone-forming osteoblasts and bone-resorbing osteoclasts which helps maintain balanced calcium homeostasis while at the same time ensuring that the skeleton is able to deliver stable mechanical support. Disruptions in this delicate equilibrium, particularly when bone resorption outpaces formation, culminate in pathology, which includes diminished bone mass and structural deterioration, a condition recognized as osteoporosis (17). However, the molecular link between childhood trauma and osteoporosis/bone damage has not been studied so far. Of note, considering that the association between circulating miRNAs and static bone microstructure is weak, and the dynamic changes in bone turnover are better reflected at the level of circulating miRNAs (18), the bone turnover markers were used in the present research to understand the bone metabolism. These markers were selected for the following reasons: procollagen type 1 amino-terminal propeptide (P1NP) is released during the synthesis of type I collagen and is regarded as an indicator of bone formation (19); elevated P1NP levels suggest increased bone formation. Osteocalcin is expressed in the mature stage of osteoblasts and released from the bone matrix during resorption (20); hence, increased osteocalcin levels refer to high bone turnover status with both increased bone formation and resorption (21). The collagen fragment β-CrossLaps (CTx) is generated during the bone resorption process (22). Increased CTx levels are indicative of enhanced bone resorption.

It has become increasingly apparent that extracellular vesicles (EVs) are important in cellular communication, leading to a crucial role in many physiological and pathological processes. These vesicles, released by cells into the extracellular environment, are involved in intercellular communication either by acting on cells surrounding the secreting cells or distant cells through the circulation of biofluids (23). Analyzing the cargo of EVs, such as microRNAs (miRNAs), can provide insights into paracrine and endocrine cell regulation and may highlight a link between childhood trauma and the impairment of bone health (24). MiRNAs regulate subsets of targeted messenger RNAs (mRNAs) (25), and are considered as part of epigenetics. There is increasing evidence that dysregulated miRNAs (in biofluids/tissues as well as in EVs) play an essential role in the pathological process of various mental disorders (26–28). In terms of childhood trauma, studies found altered expression levels of miRNAs in different tissues from childhood trauma participants (29, 30). The association between circulating miRNAs and childhood trauma was also identified; for example, a significant association was found between the altered expression of plasma miR-19b-3p and childhood traumatic experiences (31). Moreover, EV miR-450a-2-3p levels are associated with scores of total childhood trauma, emotional abuse, and physical neglect (32). The diverse properties of miRNAs and the fact that their expression in EVs is altered by physiological changes such as disease states make miRNA cargo in EVs a subject of interest to research the association between childhood trauma and bone health. Many miRNA functional annotation tools today can provide more information on potential biological processes and pathways regarding miRNAs, such as DNA Intelligent Analysis (DIANA)-miRPath v4.0 (33). Thus, miRNA informatics tools were used in this study to gain a deeper understanding of the possible roles of miRNA cargo between childhood trauma and bone health.

The present research aimed to explore the association between childhood trauma and bone metabolism by studying the cargo of EVs within three steps. Firstly, it should be analyzed, whether there are differences in miRNA expression in plasma EV between childhood trauma participants and healthy controls. Secondly, the possible associations between the EV miRNAs expression and levels of circulating bone turnover markers should be examined in the same blood samples. Thirdly, altered miRNAs expression in EVs should be used to explore enriched targeted genes and pathways.

## Methods

### Participants

For this study, data from an interventional study with depressive patients (DEPREHA (34), n=208) were used, some of whom had experienced childhood trauma. The inclusion criteria were strictly defined, resulting in a homogeneous cohort with minimal confounding variables and were as follows: individuals aged 18-65 years with a diagnosis of depressive episode (ICD-10 F32.x or F33.x), dysthymia (F34.1), or adjustment disorder with prolonged depressive reaction (F43.21); Inability to work for more than 21 days in the last 12 months due to the above diagnosis. Exclusion criteria were: Pregnancy; Hormone therapy (excluding hormonal contraception); Intellectual disability (ICD-10 F70-89); Compliance with other primary diagnoses, e.g., Hormonal/endocrine metabolic disorders (diabetes mellitus, thyroid dysfunction, renal, hepatic disorders, etc.); Neurological disorders; Dementia (ICD-10 F00-F03); Psychotropic drug dependence syndrome (ICD-10 F1x.2); Schizophrenia (ICD-10 F20); Psychotic, stress, and somatoform disorders (F40-49, unless they fall within the inclusion criteria); Emotionally unstable personality disorder (ICD-10 F60.3x) and other personality and behavioral disorders (F61-F69); Acute infections; Immune system disorders; Unstable remitting addictions other than nicotine; Acute drug abuse other than nicotine. The control group consisted of healthy volunteer participants, all of them were without the diagnosis of a depressive episode or early childhood trauma. Furthermore, the same exclusion criteria as mentioned above were applied to the control group. This enabled a comparison between depressed individuals with and without childhood trauma, alongside a control group without childhood trauma.

### Ethical statement

All participants in this study were informed of the purpose and content of the study verbally and in written form, and their permission was requested to complete the questionnaire and sign the consent form to participate. The clinical investigations were conducted according to the principles of the Declaration of Helsinki. Final ethical approval was provided on (11.05.2021) from the Ethics Review Board of the University of Potsdam, Germany (number 19/2021).

### Psychometric measures

For the assessment of depressive symptoms and severity, the Beck Depression Inventory-II (BDI-II) questionnaire (35, 36) was used. The BDI is a questionnaire consisting of a 21-item self-report questionnaire that addresses current affective, cognitive, motivational, and physiological symptoms of depression. Internal consistency in the sample was Cronbach’s Alpha 0.948. The assessment of the experience, severity, and different types of childhood trauma was driven by the Childhood Trauma Screener (CTS) (37), a 5-item screening tool derived from the Childhood Trauma Questionnaire, a retrospective 28-item self-report inventory tool (38, 39). All items from CTS were referred to as “When I was growing up (age<16 years old).” These 5 items were answered, including “never true” (1), “rarely true” (2), “sometimes true” (3), “often true” (4), and “very often true” (5). These 5-items from CTS were used to assess five types of childhood maltreatment, including emotional, physical, sexual abuse, emotional, and physical neglect. In accordance with Glaesmer et al. (37), we classified participants at risk if they rated at least mild forms of childhood abuse or neglect. We additionally controlled for response bias by the 3-item Minimization-Denial subscale from the Childhood Trauma Questionnaire, and excluded participants when indicated. Cronbach’s Alpha was previously specified with 0.76 (40).

### Plasma sample collection

Participants were instructed to stay abstinent and only drink water during the last 12 hours before assessment. Participants were also instructed to avoid high amounts of coffee, tea, and certain foods (e.g., bananas, cheese, almonds, nuts, vanilla, and citrus fruits) as well as intense exercise and unscheduled medication the previous day. About 10 ml blood was drawn into the EDTA blood tubes (Sarstedt, Germany). The blood was stored at 4°C for 30 minutes and centrifuged at 1500 g for 20 minutes to isolate the platelet-poor plasma (41). After being divided into 500 µl aliquots, EDTA-plasma samples were stored at -80°C for subsequent analyses. The hemolytic blood samples were excluded from the analysis.

### EV harvest

EVs from human plasma were isolated using Systems Bioscience’s thrombin and ExoQuick solutions (SBI, USA). Plasma samples were thawed, centrifuged at 3,000 g for 15 minutes to remove cells and cell debris, incubated with 5 µl (611U/ml) Thrombin (SBI, USA) (5 µl per 500 µl of plasma, RT, 5 minutes), and then centrifuged at 10,000 rpm for 5 minutes in order to remove the fibrin pellet. The supernatants (i.e., de-fibrinated plasma) were treated with 120 µl ExoQuick (SBI, USA) for 30 min at 4°C and centrifuged at 13,000 rpm for 2 minutes to pellet the EVs.

### EV RNA isolation

The SeraMir RNA Columns (SBI, USA) were used according to the manufacturer’s instruction for EV RNA isolation. Briefly, the EV pellet was resuspended in 350 µl Lysis Buffer, mixed with 200 µl 100% Ethanol (PanReac AppliChem, Germany), transferred to the spin column and centrifuged at 13,000 rpm for 1 minute. Then, 400 µl Wash Buffer was added and the samples were centrifuged at 13,000 rpm for 1 minute. This washing step was repeated twice. Finally, the 30 µl Elution Buffer was added in the spin column and first centrifuged at 2,000 rpm for 2 minutes to load the buffer. The spin columns were centrifuged at 13,000 rpm for 1 minute to elute the EV RNA finally.

The Agilent 2100 Bioanalyzer and RNA 6000 Pico chip Kits (Agilent Technologies, USA) were used to assess the quantity and quality of isolated EV RNA. Plasma samples with insufficient EV RNA quantity (<2 ng/ml) were discarded.

### EV RNA reverse transcription and miRNA real-time quantitative PCR

5 µl total exoRNA eluted from spin column was reverse transcribed (cDNA synthesis) using SeraMir Kits (SBI, USA) according to the manufacturer’s instructions. Real-time quantitative PCR was conducted on the CFX384 Touch Real-Time PCR Detection System (Bio-Rad Laboratories, USA) using 384-well SeraMir Profiler (SBI, USA) with and 2X Maxima SYBR Green/ROX qPCR Master Mix (Thermo Scientific, USA). The protocol was as follows: 50°C/2 min, 95°C/10 min, 40 cycles; 95°C/15 s, 60°C/1 min; data read at 60°C/1 min. The global mean normalization was applied for calculate the miRNA expression levels to reduce the influence of batch effects.

### Bone markers measurement

Measurements of P1NP, osteocalcin, and CTx were conducted directly on plasma samples with electrochemiluminescence immunoassays “ECLIA” from Roche COBAS Elecsys 2010 MODULARANALYTICS E170 according to the manufacturer’s protocol (REF 12149133 122 for osteocalcin, REF 03141071 190 for P1NP and REF 11972308122 for CTx, F. Hoffmann-La Roche, Ltd., Basel, Switzerland).

### Predicting the target genes of differently expressed miRNAs

The target genes of differently expressed miRNAs were predicted based on databases Targetscan (42), miRtarbase (43), and miRDB (44). To minimize the false positive rate and enhance result reliability, only the overlap of the predicted genes from the three databases was selected by Venn plot. The results from three databases were then imported into the Cytoscape software for constructing and visualizing the miRNA-mRNA interaction network. In Cytoscape, miRNA and mRNAs were represented as nodes, and the edges between nodes illustrated the interactions between miRNAs or mRNAs. The key mRNAs with the most interconnections, extracted using cytoHubba (version 0.1) (45), tend to highly connected to multiple miRNAs and virtual nodes in biological networks.

### Enriched biological pathway analysis

To better understand the childhood trauma related biological process, we performed enriched biological pathway analysis for the altered EV miRNAs. DIANA-miRPath v4.0 (33) software was used to identify the enriched pathways by both up-regulated and down-regulated miRNAs between CTS and healthy controls. This software identifies the targeted biological pathways via the “Kyoto Encyclopedia of Genes and Genomes (KEGG)”, providing a systematic analysis of miRNA functions, connecting genomic data with functional annotations. KEGG PATHWAY database (https://www.genome.jp/kegg/pathway.html) was applied to classify the category of miRNA-related pathways. The “pathways union” option of the miRPath software was performed. Given the database bias toward cancer/tumor-related pathways, these were excluded from the analysis for providing a more balanced assessment regarding mental health disorders.

### Statistical analysis

Due to the non-normal distribution of continuous variables such as age and body mass index (BMI) differences between groups were assessed using the Mann-Whitney U test, with results presented as median (interquartile range, IQR). Categorical variables (sex, smoking, alcohol use) were analyzed using the Chi-Square test, with results reported as frequencies.

For the first study objective, real-time PCR data on EV miRNA expression levels were analyzed using qbase+ software. The expression of miRNAs in plasma EVs from childhood trauma patients was calculated relative to expression of miRNAs in plasma EVs from healthy controls, using the 2^−∆∆Ct^ method. And the global mean normalization in qbase+ minimized technical variability, allowing for more accurate comparisons of gene expression levels across samples. The non-parametric Mann-Whitney U-test was used for differential miRNA expression analysis. The adjusted *p*-values were calculated using the FDR multiple comparison methods based on Benjamini and Hochberg methods (46). Criteria thresholds for significantly differential miRNA expression between the childhood trauma participants and healthy controls were set as adjusted *p*-values (FDR) < 0.05 and log_2_ fold change > 2 or < -2. As generated by the qbase+, bar graphs display the mean and 95% confidence intervals for comparing the miRNA expression levels in each group.

For the second objective, general linear regression models were employed to investigate the associations between specific EV miRNA and circulating bone markers and presented in three different models: model 1 was unadjusted regarding possible confounder variables. Given that age and sex impact circulating miRNAs (47, 48), and EV cargoes (49), model 2 was adjusted for age and sex. As our CTS group was combined with depression (depressive patients without CTS was excluded due to small sample size for statistical analysis), we adjusted in model 3 for the BDI-II score for controlling the influence of depression. The analysis was conducted using the IBM SPSS Statistics program (IBM SPSS 23.0).

For the third study objective regarding pathway exploration, *P*-values were obtained by the Fisher’s exact test as an enrichment analysis method, and the FDR was estimated.

## Results

The miRNA analysis was conducted in a total of n= 45 participants, but finally data of n=19 persons could analyze and are presented here due to a low detectable miRNA amount in EVs. Depressive patients with CTS, n=6; depressive patients without CTS, n=4; healthy individuals without CTS, n=9. Since the small size of the subgroup of depressive patients without CTS, this group was excluded from further analyses. And a descriptive overview is provided in the **Supplementary Table S1**.

### First step: analysis of differentially expressed miRNA in plasma EVs

The expression of 380 miRNAs was analyzed in plasma samples from *n* = 6 people with childhood trauma (age: *Median* = 50.00 (*IQR* = 22.00), BDI: *Median* = 21.00 (*IQR* = 23.00), female: 66.7%) and compared to *n* = 9 people without childhood trauma as controls (age: *Median* = 28.00 (*IQR* = 15.00), BDI: *Median* = 5.00 (*IQR* = 10.00), female: 55.6%). The two groups differ descriptively but not statically significant in basic characteristics such as age, gender or BMI (*P*-value > 0.05) (for further sample characteristics see **Table 1**).

**Table 1:** Demographic and clinical characteristic of childhood trauma subjects and healthy controls.

Variables	*N*	All (IQR)	*N*	CTS (IQR)	*N*	Controls (IQR)	Mann-Whitney U	Chi-Square	*p*-value
Sex (M/F)	15	6/9	6	2/4	9	4/5		0.185	0.667
Age (years)	15	30.00 (25.00)	6	50.00 (22.00)	9	28.00 (15.00)	11.5		0.066
Weight (Kg)	15	67.00 (14.00)	6	70.30 (24.42)	9	66.00 (9.00)	16.5		0.224
Height (cm)	15	170.00 (16.00)	6	166.50 (18.00)	9	173.00 (10.50)	20.5		0.456
BMI (kg/m^2^)	15	22.60 (5.60)	6	25.88 (5.40)	9	20.70 (2.35)	13		0.113
Smoking (no/yes)	15	14/1	6	5/1	9	9/0		1.607	0.205
Alcohol (no/yes)	15	9/6	6	5/1	9	4/5		2.269	0.132
BDI	15	11.00 (15.00)	6	21.00 (23.00)	9	5.0 (10.00)	2.5		0.002
P1NP (μg/l)	14	54.90 (22.38)	6	46.10 (35.10)	8	57.75 (18.22)	35.5		0.142
Osteocalcin (ng/ml)	13	16.40 (7.40)	6	17.65 (8.75)	7	13.20 (5.40)	12		0.234
CTx (ng/ml)	14	0.57 (0.14)	6	0.48 (0.31)	8	0.58 (0.07)	36		0.142

Mann-Whitney U values are reported for comparisons of continuous variables. Chi-square values are reported for comparisons of categorical variables. BMI, Body mass index; CTS, Childhood Trauma Screener; P1NP, Procollagen type I N-terminal propeptide; CTx, β-CrossLaps; IQR, Interquartile Range; BDI, Beck Depression Inventory-II questionnaire.

**Figure 1:**
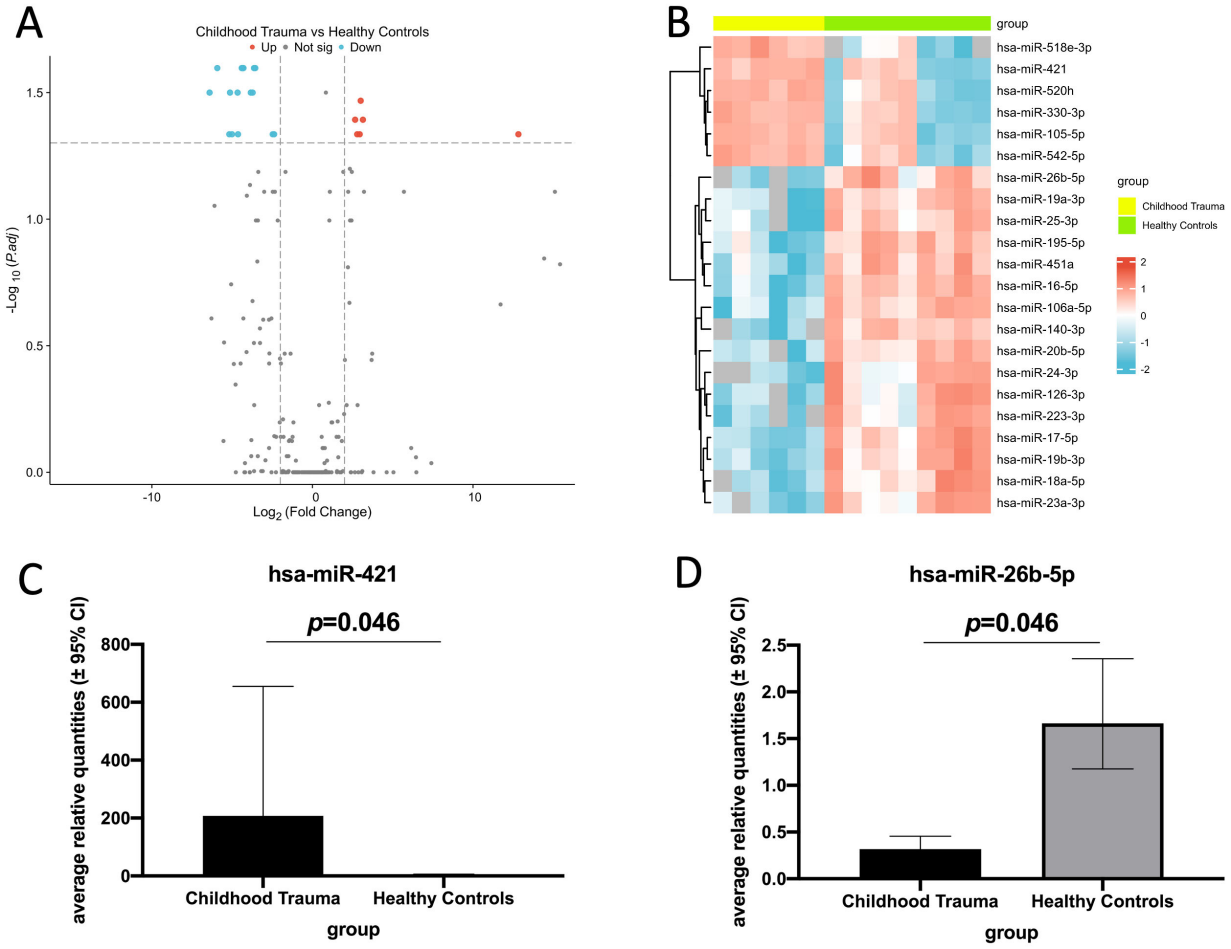
Real-time quantitative PCR analysis of differentially expressed EV miRNAs in childhood trauma participants compared to healthy controls (log_2_ fold change > 2 or < -2, adjusted p-value threshold: 0.05). **(A)** Volcano plot of all dysregulated EV miRNAs between childhood trauma and healthy controls. Blue: down-regulated expression; gray: no significant difference in the expression; red: up-regulated expression. **(B)** Heat map analysis shows all the differentially expressed EV miRNAs. Blue: down-regulated expression; red: up-regulated expression. **(C)** Bar diagram shows one up-regulated EV miRNA in childhood trauma. (hsa-miR-421, all miRNAs see **Supplementary Figure S1**). **(D)** Bar diagram shows one down-regulated EV miRNA in childhood trauma. (hsa-miR-26b-5p, all miRNAs see **Supplementary Figure S2**).

We identified 22 miRNAs that showed a significant differential expression between childhood trauma participants and healthy controls. Of these, 6 were significantly up-regulated (miR-518e-3p, miR-421, miR-520h, miR-330-3p, miR-105-5p, and miR-542-5p) and 16 were significantly down-regulated (miR-26b-5p, miR-19a-3p, miR-25-3p, miR-195-5p, miR-451a, miR-16-5p, miR-106a-5p, miR-140-3p, miR-20b-5p, miR-24-3p, miR-126-3p, miR-223-3p, miR-17-5p, miR-19b-3p, miR-18a-5p, and miR-23a-3p) in childhood trauma participants (**Figures 1A, B**). Two miRNAs (one up-regulated and one down-regulated) were showed based on their association with bone marker after adjustments (**Figures 1C, D**). The bar diagrams for all dysregulated miRNAs see **Supplementary Figures S1** and **S2**.

### Second step: the association between EV miRNAs with circulating bone markers

Results with a significant association are highlighted in **Table 2** (full association results are shown in **Supplementary Table S2**). In model 1 (unadjusted), miR-25-3p, miR-26b-5p, miR-451a, and miR-421 were associated with P1NP and CTx (all *p*-values < 0.05). Next, it was checked if age, sex and depression affect the association. The significant association of miR-25-3p and miR-451a with P1NP or CTx no longer existed in both models 2 (adjusted age and sex) and 3 (adjusted BDI), all *p*-values > 0.05, suggesting that age, sex and depression have influenced their observed association. Moreover, in model 2, miR-26b-5p positively associated with P1NP (*p*=0.029) and CTx (*p*=0.033). MiR-330-3p positively associated with osteocalcin (*p*=0.044). MiR-421 negatively associated with P1NP (*p*=0.007), and miR-542-5p positively associated with osteocalcin (*p*=0.038). In model 3, miR-26b-5p positively associated with P1NP (*p*=0.039), osteocalcin (*p*=0.037), and CTx (*p*=0.022). MiR-421 negatively associated with P1NP (*p*=0.006) and CTx (*p*=0.03).

**Table 2:** Association of EV miRNA expression levels with bone marker levels.

		Model 1^a^		Model 2^b^		Model 3^c^	
Linear regression (Listwise)	*N*	Regression coefficient	*p*-value	Adj. regression coefficient	*p*-value	Adj. regression coefficient	*p*-value
hsa-miR-25-3p							
P1NP	13	**12.261**	0.025*	9.127	0.082	12.658	0.092
osteocalcin	12	0.481	0.815	-1.201	0.603	1.572	0.577
CTx	13	**0.116**	0.021*	0.088	0.075	0.118	0.086
hsa-miR-26b-5p							
P1NP	12	**13.838**	0.009**	**14.095**	0.029*	**13.348**	0.039*
osteocalcin	11	2.051	0.146	1.332	0.449	**3.651**	0.037*
CTx	12	**0.138**	0.005**	**0.143**	0.033*	**0.139**	0.022*
hsa-miR-451a							
P1NP	14	**5.618**	0.049*	3.395	0.234	5.107	0.217
osteocalcin	13	-0.246	0.795	-1.051	0.307	0.45	0.746
CTx	14	**0.055**	0.036*	0.038	0.166	0.068	0.078
hsa-miR-330-3p							
P1NP	14	-2.817	0.45	-0.975	0.771	-0.344	0.933
osteocalcin	13	1.657	0.158	**2.275**	0.044*	1.574	0.239
CTx	14	-0.04	0.231	-0.026	0.42	-0.29	0.457
hsa-miR-421							
P1NP	14	**-0.069**	0.001**	**-0.055**	0.007**	**-0.67**	0.006**
osteocalcin	13	-0.003	0.69	0.002	0.838	-0.003	0.69
CTx	14	**-0.001**	0.011*	0	0.057	**-0.001**	0.03*
hsa-miR-542-5p							
P1NP	14	-1.569	0.621	-0.459	0.869	0.885	0.799
osteocalcin	13	1.594	0.101	**1.925**	0.038*	1.581	0.158
CTx	14	-0.027	0.35	-0.018	0.499	-0.015	0.661

a Model 1 was not adjusted.

b Model 2 was adjusted for age and sex.

c Model 3 was adjusted for BDI.

Significant Regression coefficients are bold (p<0.01, p<0.05, two sided testing); significance level: **P<0.01, *P<0.05

### Third step: exploration of the predicted target genes of differentially expressed miRNAs

**Figure 2:**
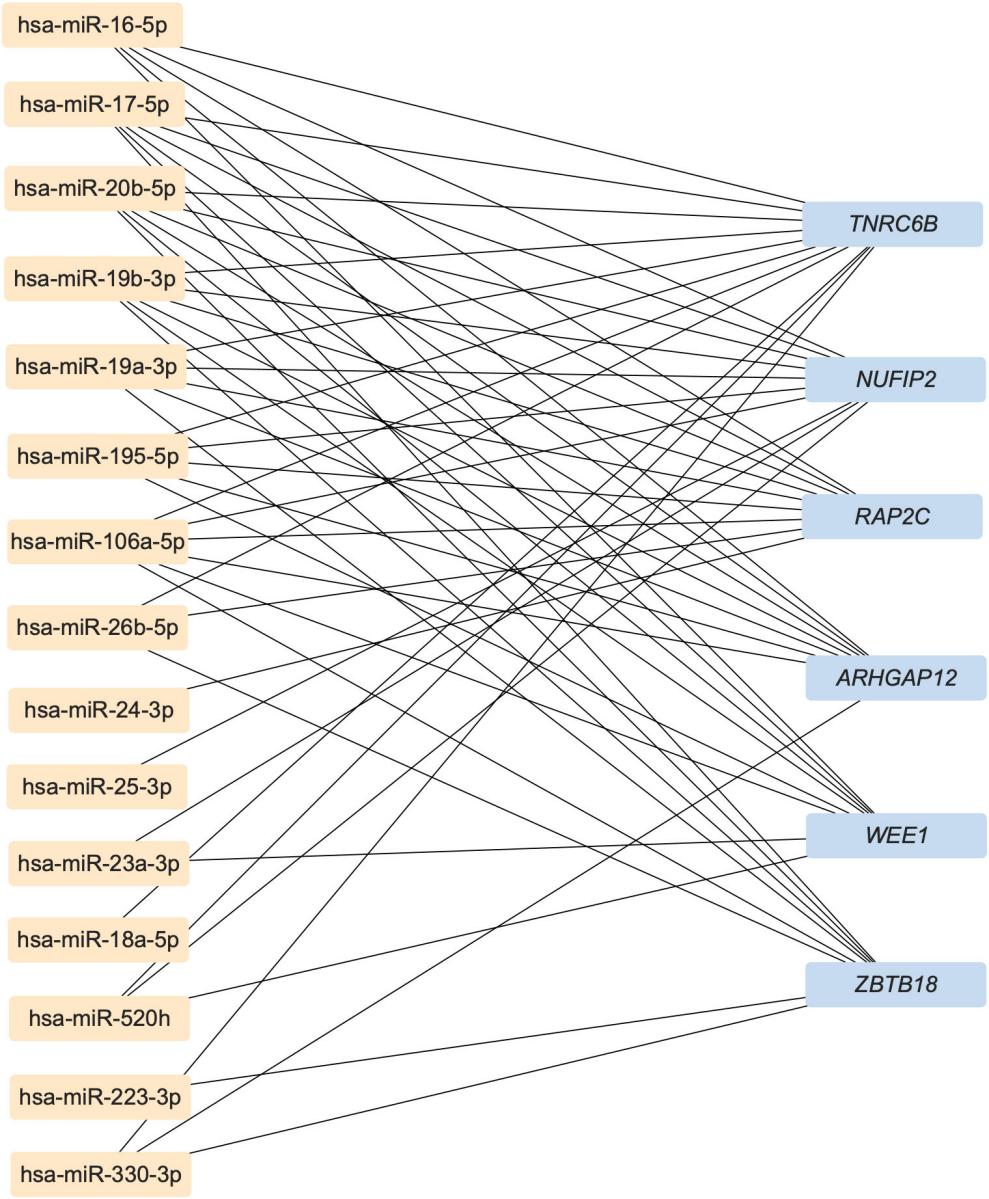
The most associated target mRNAs of these differently expressed miRNAs in childhood trauma group. TNRC6B, Trinucleotide Repeat Containing Adaptor 6B; NUFIP2, Nuclear FMR1 Interacting Protein 2; PAP2C, Phospholipid Phosphatase 2; ARHGAP12, Rho GTPase Activating Protein 12; WEE1, WEE1 G2 Checkpoint Kinase; ZBTB18, Zinc Finger And BTB Domain Containing 18.

In order to predict the highly associated target mRNAs of those altered miRNAs and further explore their potential role in bone metabolism, the miRNA-mRNA interaction network was constructed based on the target gene prediction of Targetscan, miRtarbase, and miRDB by Cytoscape (**Supplementary Figure S3**). According to the prediction, the most associated target mRNAs of these differentially expressed miRNAs in childhood trauma group, in order, were Trinucleotide Repeat Containing Adaptor 6B (*TNRC6B*), Nuclear FMR1 Interacting Protein 2 (*NUFIP2*), Phospholipid Phosphatase 2 (*PAP2C*), Rho GTPase Activating Protein 12 (*ARHGAP12*), WEE1 G2 Checkpoint Kinase (*WEE1*), and Zinc Finger And BTB Domain Containing 18 (*ZBTB18*), shown in **Figure 2**. Among, these *TNRC6B* (50), *NUFIP2* (51, 52), *ARHGAP12* (53), and *ZBTB18* (54) are known to be involved in bone metabolism.

### Third step: enriched pathways identified by DIANA-miRPath software

A total of 166 significantly enriched pathways of 22 dysregulated miRNAs with adjusted *p*-value <0.05 was explored (**Supplementary Figure S4**), and the top 10 enriched pathways based on miRNA numbers and adjusted *p*-value was showed in **Supplementary Table S3**. According to the KEGG pathway results, five pathways were Cellular Processes-related (three were associated with Cell growth and death, one was associated with Cellular community - eukaryotes, and one was associated with Transport and catabolism). Two pathways were Environmental Information Processing-related (forkhead box protein O (FoxO) and Phosphatidylinositol-4,5-bisphosphate 3-kinase (PI3K)/protein kinase B (Akt) signaling pathway, both were associated with signal transduction), one pathway was Human Diseases-related (Infectious disease: bacterial), one pathway was Organismal Systems-related (Nervous system), and one pathway was Genetic Information Processing-related (Folding, sorting and degradation).

## Discussion

This study explored the association between childhood trauma and bone metabolism through EVs, specifically their miRNA cargo. In total, 22 differentially expressed EV miRNAs were identified between childhood trauma participants and healthy controls. Among them, Van der Auwera et al. (55) found that miR-26b-5p is negatively associated with CTS score, and a significant association between altered miR-19b-3p expression and childhood traumatic experiences in bipolar depression was found (31).

Regarding the associations between those altered EV miRNAs and bone turnover markers, miR-26b-5p and miR-421 showed associations with bone turnover markers in multiple models. Therefore, a more detailed literature search was conducted to determine the possible biological role of miR-26b-5p and miR-421. Previous research showed that miR-26b-5p can alter neurite growth and synaptogenesis by targeting methyl-CpG-binding protein-2 in mouse neural stem cells (56). Therefore, miR-26b-5p may also have the potential to influence structural and functional deficits in the developing brain and thus may involve in childhood trauma processes. In addition, miR-421 is involved in regulating plasminogen activator inhibitor-1 (57), which is known to induce neuronal apoptosis (58), suggesting a possible role of miR-421 in the long-lasting process of brain development. As we aim to explore whether EVs altered due to childhood trauma may impact bone metabolism, the role of miR-26b-5p and miR-421 in bone tissue homeostasis and remodeling is of specific relevance to us. MiR-26b-5p was found up-regulated during osteogenic differentiation, and functional analysis showed that miR-26b-5p positively regulates osteogenic differentiation (59), which may explain the decrease in miR-26b-5p level found in bone samples from osteoporosis patients (60). Thus, the down-regulated miR-26b-5p can potentially affect bone tissue by impacting osteogenic differentiation. MiR-421 was found to have the ability to inhibit osteogenic differentiation of pre-osteoblasts, and an up-regulated miR-421 expression in bone tissue of osteoporosis patients was found (61). Considering these data in the context of our findings, we assume that childhood trauma may lead to reduced miR-26b-5p and increased miR-421 levels in circulating EVs, which reach bone tissues via the circulation and both lead to a weakened osteogenic differentiation, resulting in a net-decrease of bone formation.

Our results also showed that miR-26b-5p, which was down-regulated in childhood trauma participants, was positively associated with P1NP and CTx. Furthermore, miR-421, which was up-regulated in childhood trauma participants, was negatively associated with P1NP and CTx. Both suggest a reduced bone turnover (both bone formation and resorption) due to childhood trauma. It should be noted that the association between miR-26b-5p and osteocalcin became significant (*p* = 0.037) after BDI-II score adjustment in model 3, which may give notice that the direct relationship between miR-26b-5p and osteocalcin was isolated by controlling for this confounding factor (depression). This also suggests the repression of bone turnover in the context of childhood trauma. All demonstrated that altered circulating EV miRNAs due to childhood trauma could deliver specific, detrimental information on bone metabolism.

**Figure 3:**
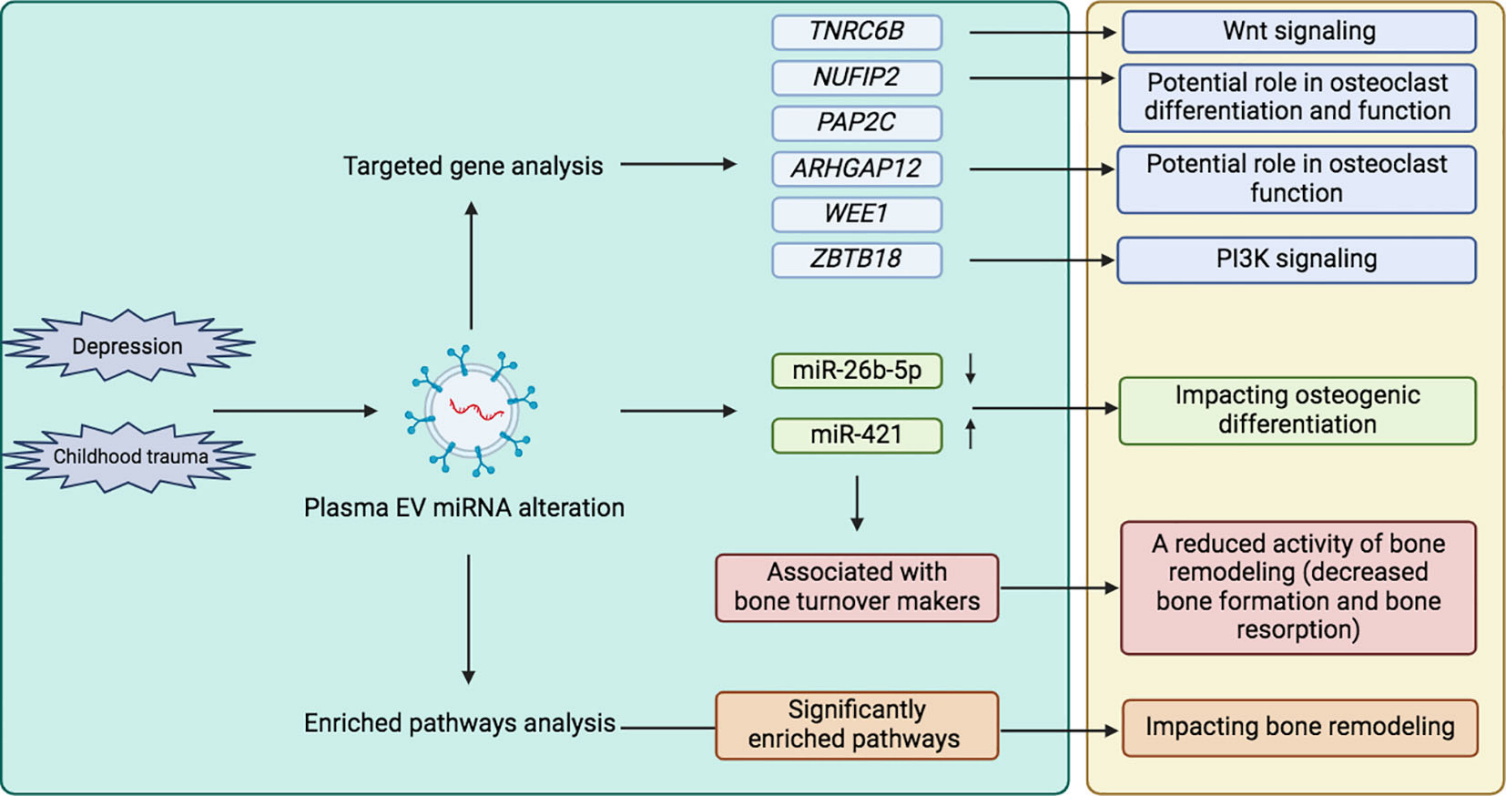
Exploring the association between childhood trauma and bone metabolism through EVs. (left green box: our research, right yellow box: combined other research from literature review). TNRC6B, Trinucleotide Repeat Containing Adaptor 6B; NUFIP2, Nuclear FMR1 Interacting Protein 2; PAP2C, Phospholipid Phosphatase 2; ARHGAP12, Rho GTPase Activating Protein 12; WEE1, WEE1 G2 Checkpoint Kinase; ZBTB18, Zinc Finger And BTB Domain Containing 18; EV, Extracellular vesicle; micro RNA, miR.

Regarding the exploration of target genes of those altered EV miRNAs, *TNRC6B* has to be highlighted due to its possible contribution to bone physiology. A single-nucleotide polymorphism in the *TNRC6B* gene has been identified as being associated with a lower spine bone mineral density and increased risk of fractures (50). The *TNRC6B* gene is also involved in bone physiology-related pathways (62), such as the Wnt signaling pathway (63). Furthermore, previous research showed the role of other observed target genes (*NUFIP2*, *ARHGAP12*, and *ZBTB18)* in bone metabolism (51–54), which showed a prospective network in the pathophysiology of childhood trauma leading to dysregulated bone metabolism (summarized in **Figure 3**). Considered those enriched pathways of miRNAs, FoxOs may be regulated by serotonin or norepinephrine signaling and the HPA axis, both are associated with stress development (64). FoxOs are also crucial in governing an array of critical functions in bone cells; for example, FoxOs promote osteogenesis and suppress osteoclastogenesis or adipogenesis by reducing levels of reactive oxygen species (65). Numerous studies have demonstrated the function of PI3K in synaptic plasticity, memory consolidation, and major depression (66, 67). Moreover, the various roles of the PI3K/Akt signaling pathway in bone metabolism have also been recognized (68). In summary, both FoxO and PI3K/Akt signaling pathways may involved in the process of childhood trauma leading to dysregulated bone metabolism. Overall, those bioinformatic analyses provided additional information for our experimental conclusions. It’s important to note that the use of miRNA informatics tools in our study is only for functional annotation purposes, and they are employed to fill the gap in the roles of miRNA, instead of conducting any form of data validation.

Nowadays, there has been a growing interest in the research of EVs and their cargo, considering the ability of miRNAs to be efficiently transfected in cells, and the epigenetic control mechanism of miRNA-mediated silencing of mRNAs make miRNAs a key research topic among EVs-carrying cargoes (69). Some differentially expressed circulating EV miRNAs due to childhood trauma have been reported (32), but the potential for EV-based pathophysiological interaction between childhood trauma and other diseases has not been elucidated. The present study focused on differentially expressed EV miRNAs and their association with bone markers in cases of childhood trauma, thus exploring the potential relationship between childhood trauma and bone metabolism via EVs.

### Strengths and limitations of the experimental study

Our sample size is relatively small because a highly controlled patient inclusion was used. We have controlled multiple variables that might affect the robustness of this research; for example, demographic and clinical characteristic variables (e.g., age, gender, BMI) were controlled between case and controls. Some variables were adjusted (age, gender, BDI) in the linear regression to control the effects on the association between miRNAs and bone markers. In a future analysis of a larger sample set, other possible influencing covariates, such as medication, smoking, alcohol, and BMI, could be considered. And while the use of model 3 (BDI-II score adjustment) helps isolate the role of depression in the association between ELS and circulating biomarkers, it does not directly address the question regarding the effects of depression on miRNA expression. Further studies compare miRNA alterations in depression, with and without ELS, would provide more insights into these associations.

Despite our efforts to match the clinical characteristics between the childhood trauma participants and healthy controls, control group participants tended to be overall younger (n=0.066). To investigate the potential impact of age differences on our results, we conducted a sub-analysis comparing the three oldest and three youngest participants in both groups, which showed no significant differences in bone biomarkers between these age subgroups, suggesting that age is unlikely to be a major confounding factor in our study. Nevertheless, to account for any potential confounding effects of age, we included age as a covariate in our regression models (model 2).

Moreover, participants were asked to avoid specific beverages and foods that are known to influence blood chemistry, and depressed patients in clinic have followed the nutrition protocol, thus reducing the influence of nutritional status. The rigorous selection process was applied to collect a highly controlled cohort, thus minimizing the impact of confounding variables and enhancing the internal validity of our findings. Furthermore, as bone mineral density test is an important indicator of bone health, we performed this test on a subgroup of study participants throughout the DEPEHA project. However, due to a limited quantity of EV RNA in some plasma samples (<2 ng/ml), the sample size for comparing bone mineral density was reduced. Thus, the direct comparison between childhood trauma participants and controls was not included in this analysis. A subgroup of participants with depression but without CTS was initially included to better investigate the effects of childhood trauma on bones; however, this group was excluded from the analysis due to its limited size.

As the enriched target gene exploration was based on database predictions, and supplementary bioinformatic analysis tool derived from online software, additional functional and mechanistic studies should be conducted in the future to substantiate this potential molecular link between childhood trauma and bone metabolism.

## Conclusion

As an exploratory study, the present study provides a prospective investigation of the relationship between childhood trauma and skeletal disorders, and hopefully provides guidance for early preventive measures, such as stress management aimed at alleviating the long-term impact of ELS on bone health. Our experimental data showed the association between dysregulated EV miRNAs and bone turnover markers, suggesting a possible role of altered miRNAs due to childhood trauma in bone metabolism. Aligned with our experimental data, this complementary bioinformatic analysis noted the potential link between childhood trauma and bone metabolism.

## Data Availability

The raw data supporting the conclusions of this article will be made available by the authors, without undue reservation.
