# Supplementary material for: Differential expression of plasma extracellular vesicles microRNAs and exploration of their association with bone metabolism in childhood trauma participants treated in a psychosomatic clinic
Source: Front Endocrinol (Lausanne). 2025 Feb 26;16:1515910. doi: 10.3389/fendo.2025.1515910 (PMC11896875; doi:10.3389/fendo.2025.1515910)
Supplement: Supplementary file 1 [file DataSheet1.docx]

**Differential expression of plasma extracellular vesicles microRNAs and exploration of their association with bone metabolism in childhood trauma participants treated in a psychosomatic clinic**

**Yangyang He ^1,2*^, Karin Wuertz-Kozak ^3,4^, Petra Cazzanelli ^3^, Sanne Houtenbos ^1,2^, Francisco Garcia-Carrizo ^5,6^*,* Tim J. Schulz ^5,6,7^, Pia-Maria Wippert ^1,2^**

1. Medical Sociology and Psychobiology, University of Potsdam, Potsdam, Germany
2. Faculty of Health Sciences Brandenburg, Joint Faculty of the University of Potsdam, The Brandenburg, Medical School Theodor Fontane and The Brandenburg University of Technology Cottbus—Senftenberg, Potsdam, Germany
3. Department of Biomedical Engineering, Rochester Institute of Technology, Rochester, NY, USA
4. Schoen Clinic Munich Harlaching, Spine Center, Academic Teaching Hospital and Spine Research Institute of the Paracelsus Medical University Salzburg, Munich, Germany
5. Department of Adipocyte Development and Nutrition, German Institute of Human Nutrition, Potsdam- Rehbrücke, Nuthetal, Germany
6. German Center for Diabetes Research (DZD), München-Neuherberg, Germany
7. Institute of Nutritional Science, University of Potsdam, Potsdam- Rehbrücke, Nuthetal, Germany

* Corresponding author


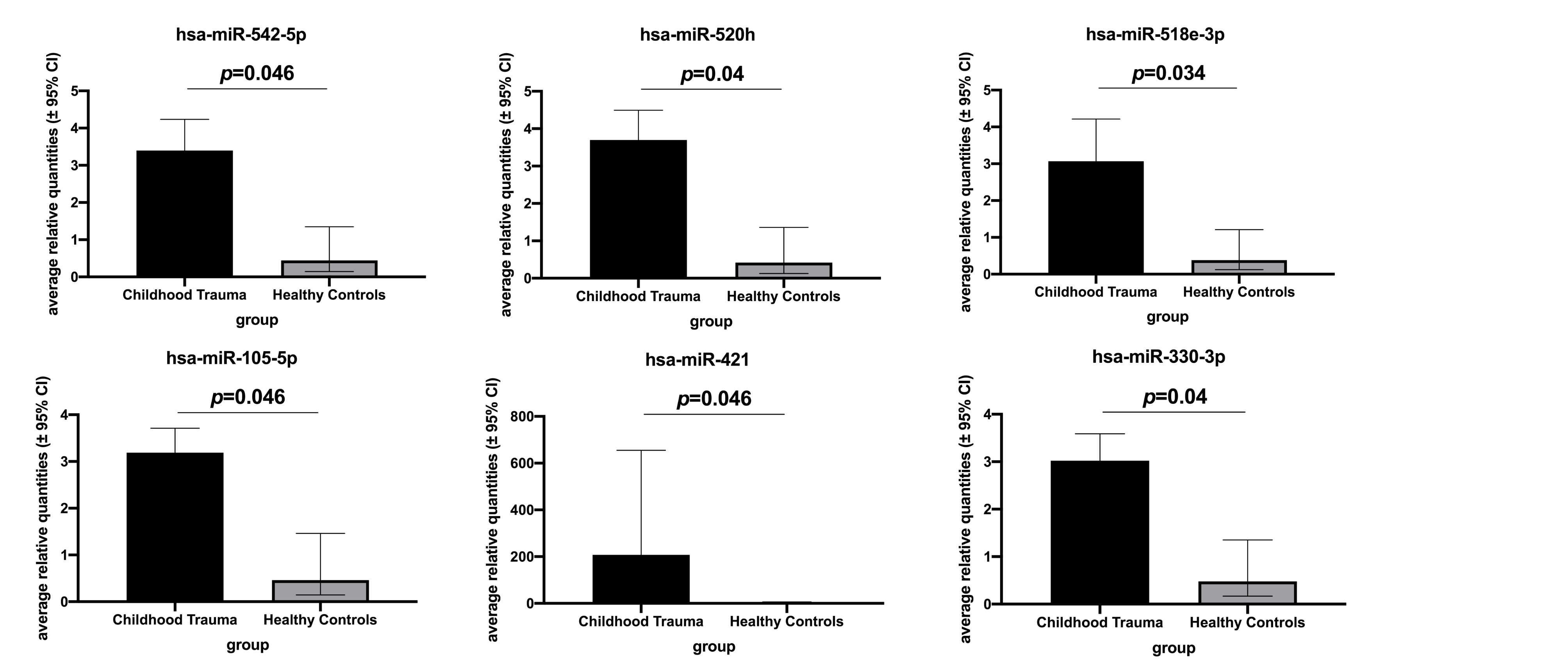


Supplementary Figure S1. Up-regulated miRNAs in childhood trauma participants. (step 1)


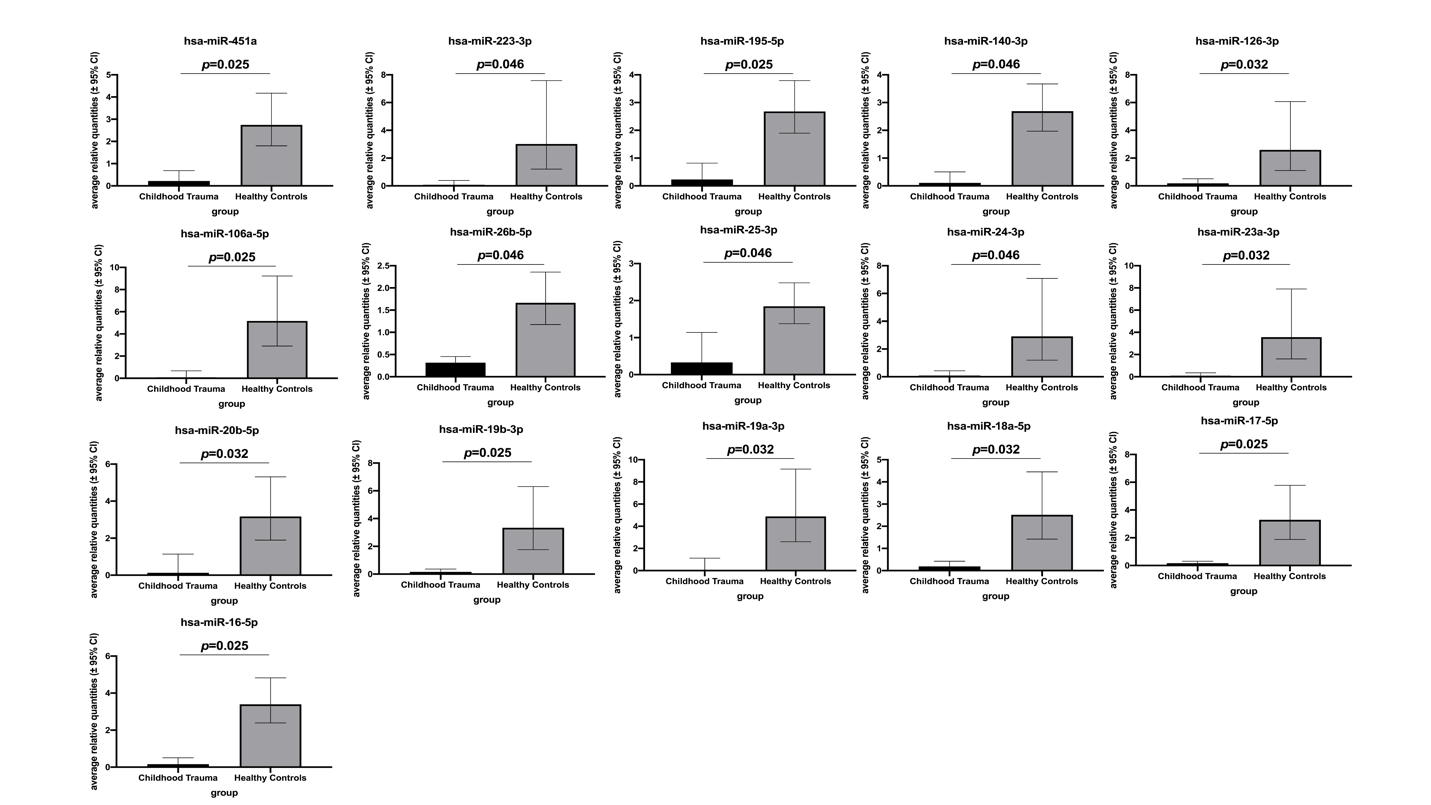


Supplementary Figure S2. Down-regulated miRNAs in childhood trauma participants. (step 1)

hsa-miR-16-5p hsa-miR-17-5p hsa-miR-18a-5p

hsa-miR-19a-3p hsa-miR-19b-3p hsa-miR-20b-5p

hsa-miR-23a-3p hsa-miR-24-3p hsa-miR-25-3p

hsa-miR-26b-5p hsa-miR-105-5p hsa-miR-106a-5p

hsa-miR-126-3p hsa-miR-140-3p hsa-miR-195-5p

hsa-miR-223-3p hsa-miR-330-3p hsa-miR-421

hsa-miR-451a hsa-miR-518e-3p hsa-miR-520h

hsa-miR-542-5p

Supplementary Figure S3. Predicted target gene of differentially expressed miRNAs based on Targetscan, miRDB, and miRtarbase (step 3)


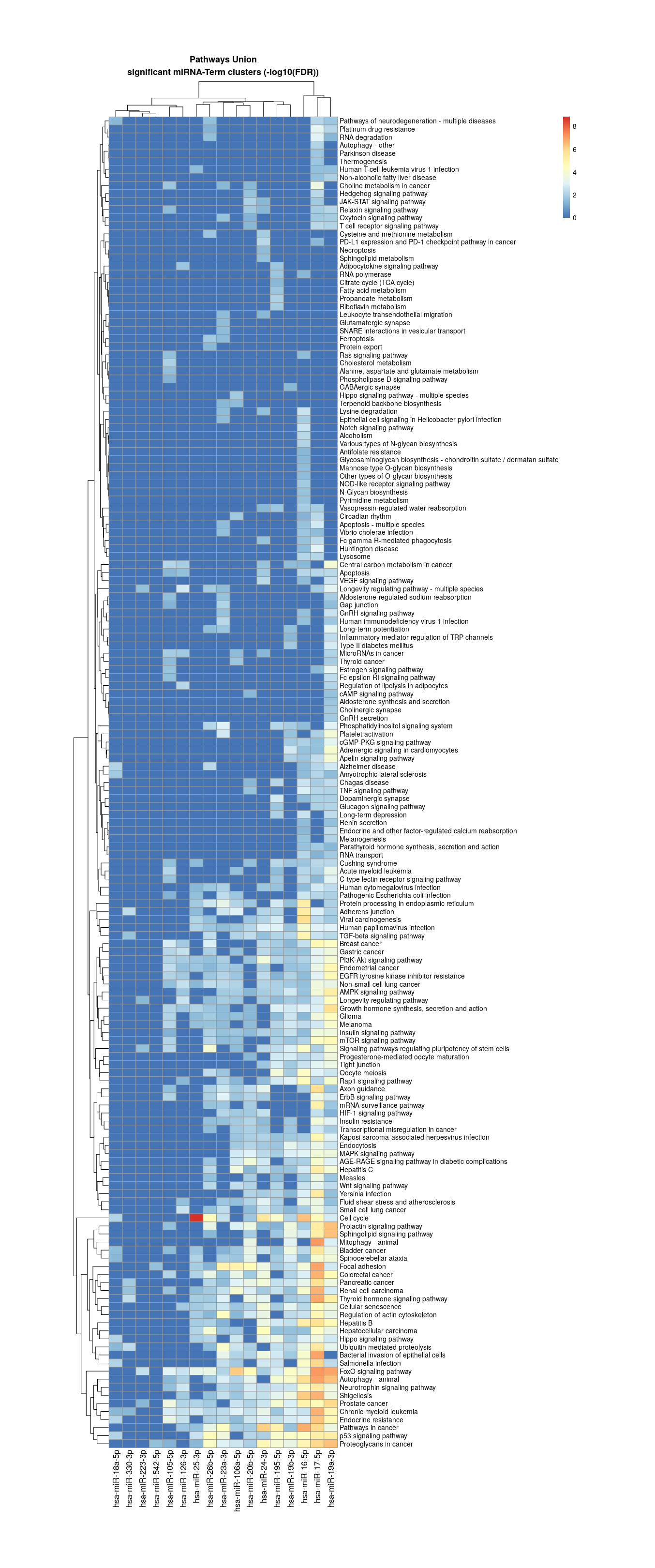


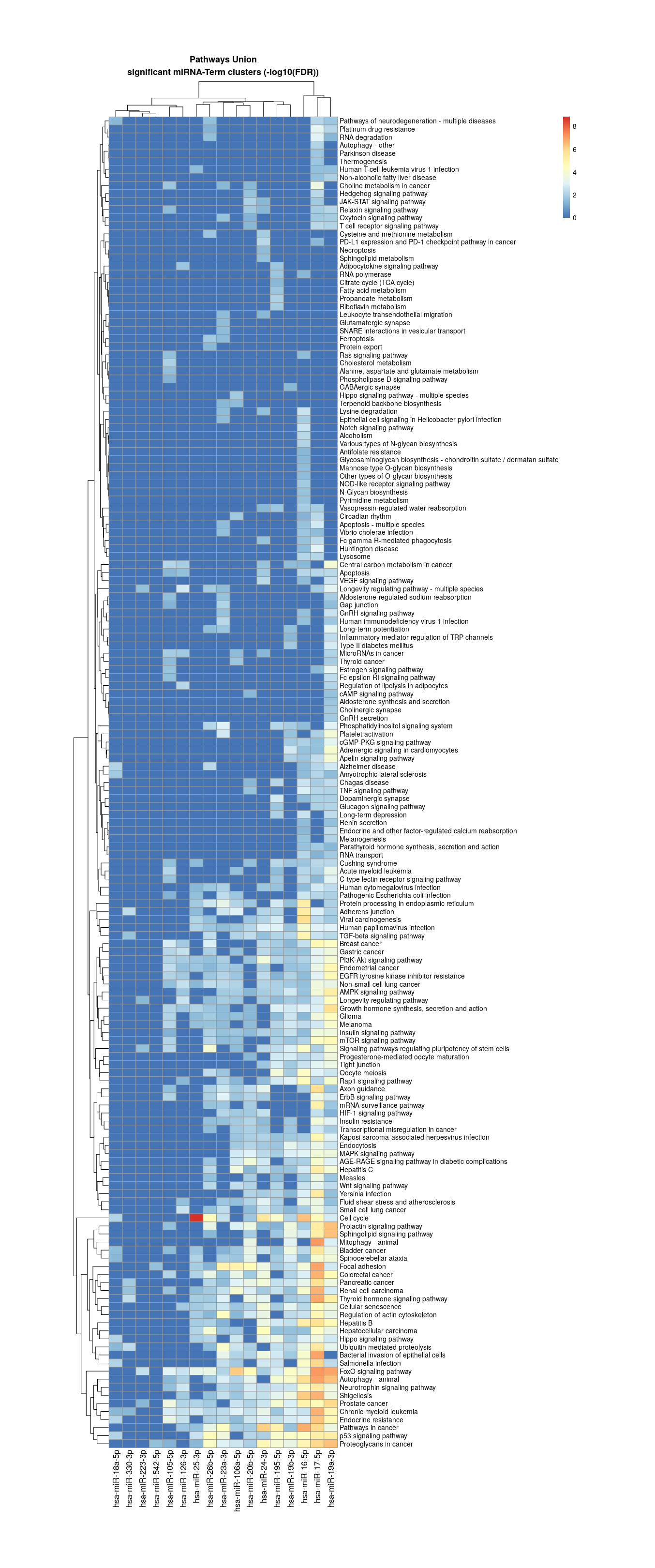
Supplementary Figure S4. Enriched pathways identified by DIANA-miRPath 4.0 software (step 3)

Supplementary Table S1. Demographic and clinical characteristic of depression subjects (with or without CTS) and healthy controls

| **Variables** | ***N*** | **All (IQR)** | ***N*** | **Depresion with CTS (IQR)** | ***N*** | **Depression without CTS (IQR)** | ***N*** | **Controls (IQR)** |
| --- | --- | --- | --- | --- | --- | --- | --- | --- |
| Sex (M/F) | 19 | 8/11 | 6 | 2/4 | 4 | 2/2 | 9 | 4/5 |
| Age (years) | 19 | 35.00 (26.00) | 6 | 50.00 (22.00) | 4 | 48.50 (24.00) | 9 | 28.00 (15.00) |
| Weight (Kg) | 19 | 68.00 (23.00) | 6 | 70.30 (24.42) | 4 | 92.30 (38.70) | 9 | 66.00 (9.00) |
| Height (cm) | 19 | 173.00 (16.00) | 6 | 166.50 (18.00) | 4 | 178.00 (20.5) | 9 | 173.00 (10.50) |
| BMI (kg/m^2^) | 19 | 24.61 (7.40) | 6 | 25.88 (5.40) | 4 | 29.10 (5.40) | 8 | 20.70 (2.35) |
| Smoking (no/yes) | 19 | 17/2 | 6 | 5/1 | 4 | 3/1 | 9 | 9/0 |
| Alcohol (no/yes) | 19 | 13/6 | 6 | 5/1 | 4 | 4/0 | 9 | 4/5 |
| BDI | 18 | 11.00 (22.00) | 6 | 21.00 (23.00) | 3 | 24.00 (.) | 9 | 5.00 (10.00) |
| P1NP (μg/l) | 18 | 54.00 (26.68) | 6 | 46.10 (35.10) | 4 | 36.85 (29.28) | 8 | 57.75 (18.22) |
| Osteocalcin (ng/ml) | 17 | 16.10 (7.20) | 6 | 17.65 (8.75) | 4 | 14.50 (9.05) | 7 | 13.20 (5.40) |
| CTx ( ng/ml) | 18 | 0.56 (0.30) | 6 | 0.48 (0.31) | 4 | 0.23 (0.38) | 8 | 0.58 (0.07) |

Supplementary Table S2. Association of EV miRNA expression levels with bone marker levels (step 2)

|  |  | **Model 1^a^** |  | **Model 2^b^** |  | **Model 3^c^** |  |
| --- | --- | --- | --- | --- | --- | --- | --- |
| **Linear regression (Listwise)** | **N** | **Regression coefficient** | **p-value** | **Adj. regression coefficient** | **p-value** | **Adj. regression coefficient** | **p-value** |
| hsa-miR-16-5p |  |  |  |  |  |  |  |
| P1NP | 14 | 4.158 | 0.114 | 2.678 | 0.266 | 2.736 | 0.489 |
| osteocalcin | 13 | -0.888 | 0.319 | -1.463 | 0.104 | -0.911 | 0.497 |
| CTX | 14 | 0.042 | 0.079 | 0.03 | 0.188 | 0.048 | 0.191 |
| hsa-miR-17-5p |  |  |  |  |  |  |  |
| P1NP | 14 | 3.275 | 0.124 | 1.841 | 0.359 | 2.09 | 0.448 |
| osteocalcin | 13 | -0.516 | 0.479 | -1.085 | 0.15 | -0.33 | 0.728 |
| CTX | 14 | 0.032 | 0.103 | 0.02 | 0.295 | 0.03 | 0.257 |
| hsa-miR-18a-5p |  |  |  |  |  |  |  |
| P1NP | 13 | 3.387 | 0.154 | 1.963 | 0.372 | 2.088 | 0.437 |
| osteocalcin | 12 | -0.306 | 0.656 | -0.723 | 0.268 | -0.079 | 0.921 |
| CTX | 13 | 0.023 | 0.332 | 0.009 | 0.708 | 0.013 | 0.632 |
| hsa-miR-19a-3p |  |  |  |  |  |  |  |
| P1NP | 13 | 2.114 | 0.139 | 1.231 | 0.371 | 1.436 | 0.418 |
| osteocalcin | 12 | -0.203 | 0.696 | -0.65 | 0.224 | -0.16 | 0.806 |
| CTX | 13 | 0.022 | 0.086 | 0.015 | 0.228 | 0.017 | 0.291 |
| hsa-miR-19b-3p |  |  |  |  |  |  |  |
| P1NP | 14 | 2.388 | 0.21 | 1.638 | 0.333 | 1.069 | 0.651 |
| osteocalcin | 13 | -0.722 | 0.31 | -1.049 | 0.136 | -0.646 | 0.473 |
| CTX | 14 | 0.023 | 0.19 | 0.017 | 0.31 | 0.017 | 0.446 |
| hsa-miR-20b-5p |  |  |  |  |  |  |  |
| P1NP | 13 | 2.188 | 0.347 | 1.783 | 0.392 | 0.49 | 0.863 |
| osteocalcin | 12 | -0.897 | 0.279 | -1.095 | 0.168 | -1.068 | 0.305 |
| CTX | 13 | 0.028 | 0.175 | 0.026 | 0.169 | 0.016 | 0.525 |
| hsa-miR-23a-3p |  |  |  |  |  |  |  |
| P1NP | 13 | 1.778 | 0.19 | 1.241 | 0.237 | 1,027 | 0.495 |
| osteocalcin | 12 | -0.355 | 0.47 | -0.589 | 0.223 | -0.268 | 0.633 |
| CTX | 13 | 0.011 | 0.386 | 0.006 | 0.605 | 0.006 | 0.686 |
| hsa-miR-24-3p |  |  |  |  |  |  |  |
| P1NP | 12 | 1.815 | 0.194 | 1.848 | 0.052 | 1.815 | 0.194 |
| osteocalcin | 11 | -0.442 | 0.389 | -0.523 | 0.212 | -0.344 | 0.563 |
| CTX | 12 | 0.011 | 0.433 | 0.01 | 0.467 | 0.005 | 0.746 |
| hsa-miR-25-3p |  |  |  |  |  |  |  |
| P1NP | 13 | **12.261** | 0.025* | 9.127 | 0.082 | 12.658 | 0.092 |
| osteocalcin | 12 | 0.481 | 0.815 | -1.201 | 0.603 | 1.572 | 0.577 |
| CTX | 13 | **0.116** | 0.021* | 0.088 | 0.075 | 0.118 | 0.086 |
| hsa-miR-26b-5p |  |  |  |  |  |  |  |
| P1NP | 12 | **13.838** | 0.009** | **14.095** | 0.029* | **13.348** | 0.039* |
| osteocalcin | 11 | 2.051 | 0.146 | 1.332 | 0.449 | **3.651** | 0.037* |
| CTX | 12 | **0.138** | 0.005** | **0.143** | 0.033* | **0.139** | 0.022* |
| hsa-miR-451a |  |  |  |  |  |  |  |
| P1NP | 14 | **5.618** | 0.049* | 3.395 | 0.234 | 5.107 | 0.217 |
| osteocalcin | 13 | -0.246 | 0.795 | -1.051 | 0.307 | 0.45 | 0.746 |
| CTX | 14 | **0.055** | 0.036* | 0.038 | 0.166 | 0.068 | 0.078 |
| hsa-miR-105-5p |  |  |  |  |  |  |  |
| P1NP | 14 | -2.841 | 0.42 | -1.539 | 0.62 | -0.14 | 0.972 |
| osteocalcin | 13 | 1.342 | 0.237 | 1.74 | 0.117 | 1.24 | 0.356 |
| CTX | 14 | -0.04 | 0.205 | -0.03 | 0.312 | -0.029 | 0.443 |
| hsa-miR-106a-5p |  |  |  |  |  |  |  |
| P1NP | 14 | 1.801 | 0.112 | 0.94 | 0.394 | 1.221 | 0.378 |
| osteocalcin | 13 | -0.227 | 0.577 | -0.63 | 0.146 | -0.106 | 0.832 |
| CTX | 14 | 0.016 | 0.133 | 0.009 | 0.424 | 0.013 | 0.316 |
| hsa-miR-126-3p |  |  |  |  |  |  |  |
| P1NP | 13 | 1.793 | 0.377 | 1.078 | 0.525 | 0.827 | 0.685 |
| osteocalcin | 12 | -0.503 | 0.45 | -0.822 | 0.205 | -0.488 | 0.516 |
| CTX | 13 | 0.017 | 0.316 | 0.012 | 0.472 | 0.008 | 0.667 |
| hsa-miR-140-3p |  |  |  |  |  |  |  |
| P1NP | 12 | 4.345 | 0.093 | 3.969 | 0.145 | 0.028 | 0.583 |
| osteocalcin | 11 | -0.554 | 0.575 | -0.748 | 0.512 | 0.105 | 0.958 |
| CTX | 12 | 0.041 | 0.113 | 0.037 | 0.202 | 0.028 | 0.583 |
| hsa-miR-195-5p |  |  |  |  |  |  |  |
| P1NP | 14 | 4.988 | 0.092 | 3.188 | 0.252 | 3.684 | 0.386 |
| osteocalcin | 13 | -0.528 | 0.574 | -1.075 | 0.266 | -0.077 | 0.959 |
| CTX | 14 | 0.049 | 0.07 | 0.035 | 0.187 | 0.055 | 0.17 |
| hsa-miR-223-3p |  |  |  |  |  |  |  |
| P1NP | 12 | 0.915 | 0.45 | 0.711 | 0.57 | -0.177 | 0.884 |
| osteocalcin | 11 | -0.462 | 0.386 | -0.654 | 0.242 | -0.487 | 0.431 |
| CTX | 12 | 0.009 | 0.433 | 0.008 | 0.525 | -0.001 | 0.932 |
| hsa-miR-330-3p |  |  |  |  |  |  |  |
| P1NP | 14 | -2.817 | 0.45 | -0.975 | 0.771 | -0.344 | 0.933 |
| osteocalcin | 13 | 1.657 | 0.158 | **2.275** | 0.044* | 1.574 | 0.239 |
| CTX | 14 | -0.04 | 0.231 | -0.026 | 0.42 | -0.29 | 0.457 |
| hsa-miR-421 |  |  |  |  |  |  |  |
| P1NP | 14 | **-0.069** | 0.001** | **-0.055** | 0.007** | **-0.67** | 0.006** |
| osteocalcin | 13 | -0.003 | 0.69 | 0.002 | 0.838 | -0.003 | 0.69 |
| CTX | 14 | **-0.001** | 0.011* | 0 | 0.057 | **-0.001** | 0.03* |
| hsa-miR-518e-3p |  |  |  |  |  |  |  |
| P1NP | 12 | -3.336 | 0.375 | -2.382 | 0.472 | 0.14 | 0.98 |
| osteocalcin | 12 | 0.776 | 0.476 | 0.987 | 0.375 | 0.61 | 0.718 |
| CTX | 12 | -0.054 | 0.103 | -0.048 | 0.108 | -0.067 | 0.186 |
| hsa-miR-520h |  |  |  |  |  |  |  |
| P1NP | 14 | -4.542 | 0.108 | -3.067 | 0.231 | -3.13 | 0.36 |
| osteocalcin | 13 | 0.498 | 0.604 | 0.972 | 0.322 | 0.202 | 0.863 |
| CTX | 14 | -0.047 | 0.064 | -0.036 | 0.144 | -0.045 | 0.16 |
| hsa-miR-542-5p |  |  |  |  |  |  |  |
| P1NP | 14 | -1.569 | 0.621 | -0.459 | 0.869 | 0.885 | 0.799 |
| osteocalcin | 13 | 1.594 | 0.101 | **1.925** | 0.038* | 1.581 | 0.158 |
| CTX | 14 | -0.027 | 0.35 | -0.018 | 0.499 | -0.015 | 0.661 |

^a^ Model 1 was not adjusted.

^b^ Model 2 was adjusted for age and sex.

^c^ Model 3 was adjusted for BDI.

Significant Regression coefficients are bold (p <0.01, p <0.05, two sided testing); significance level: **P<0.01, *P<0.05

Supplementary Table S3. Top 10 enriched pathways identified by DIANA-miRPath 4.0 software (step 3)

| **KEGG pathway maps** | **Term Name** | **miRNAs** | **miRNA Names** | **Merged FDR** |
| --- | --- | --- | --- | --- |
| Environmental Information Processing; Signal transduction | FoxO signaling pathway | 14 | hsa-miR-16-5p,hsa-miR-17-5p,hsa-miR-19a-3p,hsa-miR-19b-3p,hsa-miR-23a-3p,hsa-miR-24-3p,hsa-miR-25-3p,hsa-miR-26b-5p,hsa-miR-105-5p,hsa-miR-106a-5p,hsa-miR-223-3p,hsa-miR-126-3p,hsa-miR-195-5p,hsa-miR-20b-5p | 9.54796^-60^ |
| Cellular Processes; Cellular community - eukaryotes | Focal adhesion | 12 | hsa-miR-16-5p,hsa-miR-17-5p,hsa-miR-19a-3p,hsa-miR-19b-3p,hsa-miR-23a-3p,hsa-miR-24-3p,hsa-miR-25-3p,hsa-miR-26b-5p,hsa-miR-106a-5p,hsa-miR-195-5p,hsa-miR-20b-5p,hsa-miR-542-5p | 1.54964^-42^ |
| Cellular Processes; Cell growth and death | Cellular senescence | 12 | hsa-miR-16-5p,hsa-miR-17-5p,hsa-miR-19a-3p,hsa-miR-19b-3p,hsa-miR-23a-3p,hsa-miR-24-3p,hsa-miR-25-3p,hsa-miR-26b-5p,hsa-miR-106a-5p,hsa-miR-126-3p,hsa-miR-195-5p,hsa-miR-20b-5p | 1.1881^-30^ |
| Environmental Information Processing; Signal transduction | PI3K-Akt signaling pathway | 12 | hsa-miR-16-5p,hsa-miR-17-5p,hsa-miR-19a-3p,hsa-miR-19b-3p,hsa-miR-23a-3p,hsa-miR-24-3p,hsa-miR-25-3p,hsa-miR-26b-5p,hsa-miR-105-5p,hsa-miR-126-3p,hsa-miR-195-5p,hsa-miR-20b-5p | 1.1316^-26^ |
| Cellular Processes; Cell growth and death | Cell cycle | 11 | hsa-miR-16-5p,hsa-miR-17-5p,hsa-miR-18a-5p,hsa-miR-19a-3p,hsa-miR-19b-3p,hsa-miR-23a-3p,hsa-miR-24-3p,hsa-miR-25-3p,hsa-miR-26b-5p,hsa-miR-195-5p,hsa-miR-20b-5p | 5.51092^-47^ |
| Cellular Processes; Cell growth and death | p53 signaling pathway | 11 | hsa-miR-16-5p,hsa-miR-17-5p,hsa-miR-18a-5p,hsa-miR-19a-3p,hsa-miR-19b-3p,hsa-miR-23a-3p,hsa-miR-24-3p,hsa-miR-25-3p,hsa-miR-26b-5p,hsa-miR-195-5p,hsa-miR-20b-5p | 4.57407^-43^ |
| Cellular Processes; Transport and catabolism | Autophagy - animal | 11 | hsa-miR-16-5p,hsa-miR-17-5p,hsa-miR-19a-3p,hsa-miR-19b-3p,hsa-miR-23a-3p,hsa-miR-26b-5p,hsa-miR-105-5p,hsa-miR-106a-5p,hsa-miR-126-3p,hsa-miR-195-5p,hsa-miR-20b-5p | 2.03822^-42^ |
| Human Diseases; Infectious disease: bacterial | Shigellosis | 11 | hsa-miR-16-5p,hsa-miR-17-5p,hsa-miR-19a-3p,hsa-miR-19b-3p,hsa-miR-23a-3p,hsa-miR-24-3p,hsa-miR-26b-5p,hsa-miR-106a-5p,hsa-miR-126-3p,hsa-miR-195-5p,hsa-miR-20b-5p | 4.21899^-36^ |
| Organismal Systems; Nervous system | Neurotrophin signaling pathway | 11 | hsa-miR-16-5p,hsa-miR-17-5p,hsa-miR-19a-3p,hsa-miR-19b-3p,hsa-miR-23a-3p,hsa-miR-24-3p,hsa-miR-105-5p,hsa-miR-106a-5p,hsa-miR-126-3p,hsa-miR-195-5p,hsa-miR-20b-5p | 1.74472^-31^ |
| Genetic Information Processing; Folding, sorting and degradation | Ubiquitin mediated proteolysis | 11 | hsa-miR-16-5p,hsa-miR-17-5p,hsa-miR-18a-5p,hsa-miR-19a-3p,hsa-miR-19b-3p,hsa-miR-23a-3p,hsa-miR-26b-5p,hsa-miR-106a-5p,hsa-miR-195-5p,hsa-miR-330-3p,hsa-miR-20b-5p | 9.85383^-31^ |

KEGG: Kyoto Encyclopedia of Genes and Genomes; FoxO: forkhead box protein O; PI3K: Phosphatidylinositol-4,5-bisphosphate 3-kinase; Akt: protein kinase B; hsa-miR: *Homo sapiens* microRNA.
